# Evaluating the Effects of KCl on Thermal Behavior and Reaction Kinetics of Medium Density Fiberboard Pyrolysis

**DOI:** 10.3390/ma12111826

**Published:** 2019-06-05

**Authors:** Longwei Pan, Yong Jiang

**Affiliations:** State Key Laboratory of Fire Science, University of Science and Technology of China, Hefei 230027, China; yuanhq@mail.ustc.edu.cn

**Keywords:** pyrolysis, medium density fiberboard, potassium chloride, reaction kinetics, differential evolution, TG−FTIR

## Abstract

The effects of potassium chloride (KCl) on the pyrolysis of medium density fiberboard (MDF) were investigated by using thermogravimetry/Fourier-transfer infrared spectroscopy (TG-FTIR). Five MDF samples treated with different KCl concentrations (0%, 0.5%, 1%, 2% and 3%) were heated with a heating rate of 20 °C/min. The thermogravimetry (TG) results showed that KCl caused the primary pyrolysis stage towards lower temperatures. The FTIR results indicated that with the concentrations of KCl, the formation of CH_4_ and C=O functional groups decreased while the formation of CO_2_ and CO increased. To figure out the reason for the observed phenomena, the kinetic parameters in primary pyrolysis and the secondary charring reaction were estimated by a differential evolution (DE) optimization algorithm. The prediction indicated that KCl shifted the initial degradation temperature of each component of MDF towards a lower temperature. Char and gas yields increased with the concentration of KCl, whereas the tar yield reduced. The changes in activation energies revealed that KCl played a catalyst role in the reaction of resin, hemicellulose and cellulose in primary pyrolysis. For lignin, KCl had little effect. In the secondary charring reaction, KCl apparently promoted the reaction of tar. The catalytic effect of KCl on MDF pyrolysis was the combination of primary pyrolysis and the secondary charring reaction. Finally, the optimal catalytic concentration for KCl on MDF pyrolysis was analyzed.

## 1. Introduction

Medium density fiberboard (MDF) is extensively applied as an eco-friendly material in the fields of buildings, furniture and decoration in China. Global MDF production was more than 100 million m^3^ in 2017 and over 59 million m^3^ was produced by China [1]. However, there are some disadvantages exhibited by MDF. Firstly, the raw materials of MDF are derived from wood or other plants, which make it flammable. It is well known that solid combustion undergoes a pyrolysis process first. Secondly, a large amount of waste MDF is generated, owing to its unsteady structure. As a type of biomass, it is an ideal choice to recycle MDF waste as a renewable source [2]. Therefore, thermochemical conversion technologies are attracting increased attention due to the advantages in waste management [3,4]. Pyrolysis is a promising thermochemical technology used to transform these considerable amounts of waste into bio-oil, bio-char and gaseous products [5,6]. Thus, from the perspective of fire prevention and biomass energy recycling, it is very essential to study MDF pyrolysis characteristics and reaction kinetics.

Biomass is composed of extractives, hemicellulose, cellulose, lignin and a certain amount of inorganic species [7]. Alkali and alkali-earth metal (AAEM) present the major inorganic species in biomass, accounting for more than 85% [8]. The inorganic species have an obvious effect on the pyrolysis behavior of biomass and product yields [9,10]. A number of studies have been performed to investigate the effects of inorganic species on biomass pyrolysis. As observed by Raveendran et al., AAEM species influenced both the pyrolysis behaviors and the product yields strongly [9]. Scott et al. first conducted research into the effect of lime (CaO) on liquid products of biomass pyrolysis [11]. Nowakowski et al. suggested that the presence of potassium made the char yields increase from the short rotation willow coppice pyrolysis [12]. Shimada et al. found that potassium chloride and sodium chloride reduced the levoglucose yield, as well as promoted water, carbon monoxide and char formation [13]. As reported by Pan and Richards, potassium played a catalyst role in the process of pyrolysis reactions [14]. Patwardhan et al. compared the effects of different inorganic species on biomass pyrolysis and found that the presence of potassium and sodium reduced the yields of levoglucose, while calcium and magnesium had a much weaker effect [15]. Feng et al. observed that the gas and char yields increased with the increasing concentration of potassium and calcium, while tar yield decreased [8]. Despite these studies, the underlying reason of the observed changes in pyrolysis behaviors and product yields of biomass remains unclear. There is a necessity to figure out whether the observed changes are due to primary pyrolysis or secondary charring reactions. 

As argued by Trendewicz et al., the major AAEM found in biomass are potassium, sodium, calcium and magnesium [16], which exist mainly in water soluble salts [17]. Compared with other inorganic species, potassium has a relatively large weight fraction in biomass [18,19,20]. In this paper, potassium was selected to investigate its effect on the pyrolysis of MDF. The aim of this paper was to determine the cause of the influence of potassium on the pyrolysis of MDF. First, the thermal behavior of MDF pyrolysis was observed by heating samples with varying potassium chloride (KCl) concentrations using a thermogravimetric analyzer (TG). Then, the effects of KCl on the gaseous pyrolysis products were studied by using Fourier-transfer infrared (FTIR) spectroscopy. The differential evolution (DE) algorithm coupled with the experimental data was employed to evaluate the reaction kinetics parameters. Finally, some empirical formulas of the reactions in MDF pyrolysis process were obtained based on the analysis of the changes in activation energies caused by potassium.

## 2. Materials and Methods 

### 2.1. Material Samples

The MDF applied to performing this study was identical to that in the previous study [21]. The MDF particles sized less than 125 μm were derived from a Tyler sieve series. Proximate analysis was conducted using a TGA 701 thermogravimetric analyzer (LECO, San Jose, Michigan, USA). Ultimate analysis was performed using a TruSpec CHN (LECO, San Jose, Michigan, USA) and TruSpec S (LECO, San Jose, Michigan, USA) to detect the content of carbon, hydrogen, nitrogen and sulfur. The content of oxygen was calculated by difference. The proximate and ultimate analysis results of the MDF sample are shown in Table 1, where the values are the mean results obtained from three repeated tests.

In order to remove minerals from the MDF sample, a hot water wash procedure was applied. Firstly, 10 g of MDF was stirred with 200 ml of deionized water in a beaker for 5 h at 100 °C. Next, the washed MDF was filtrated and washed with 200 ml of deionized water again. Then, the wet sample was dehydrated in a drying oven at 100 °C for at least 24 h. The ash compositions of both raw and washed MDF samples were detected by XRF (FLUOROLOG-3-TAU, Jobin Yvon, Paris, France). All samples were tested for three times and the average results are presented in Table 2. This revealed that the content of potassium and chlorine in the ash decreased from 10.66 wt% to 1.25 wt% and from 0.53 wt% to 0.20 wt%, respectively. Thus, the removal rates of potassium and chlorine were 94.2% and 81.3% by water washing, respectively, since the ash content of the washed sample was about 2.32%.

To add potassium to the washed MDF sample, the sample was impregnated with KCl. In this work, based on the typical range of the potassium content in biomass [22,23], the washed MDF was doped with four KCl concentrations of 0.5, 1, 2 and 3 wt%. Taking 1 wt% loading concentration of KCl in the washed sample as an example, 0.005 g of KCl and 0.495 g washed sample were first added into 3 ml of deionized water in a beaker. Secondly, the mixture was stirred for 12 h to distribute the potassium salt uniformly. Then, the solution was dehydrated in an oven at 100 °C for at least 24 h. Finally, a total of five MDF samples with 0, 0.5, 1, 2 and 3 wt% KCl were derived.

### 2.2. TG-FTIR

The pyrolysis of samples were carried out using a TG-FTIR system comprised of a NETZSCH STA 449 F3 Jupiter (NETZSCH, Selb, Germany) thermal analyzer and a Fourier transform infrared (FTIR) spectrometer (PerkinElmer Frontier, Waltham, Massachusetts, USA). The samples of 8 ± 0.5 mg were heated from 30 to 800 °C at a heating rate of 20 °C/min with a gas flow of 80 ml/min under a nitrogen atmosphere. Aside from this, the transfer pipe connecting the TG analyzer and FTIR was heated to a constant temperature of 280 °C to minimize the condensation of gases during the pyrolysis process. FTIR spectra were recorded from 4000 to 450 cm^−1^ with a scan frequency of 20 times per minute to identify the composition of the gas products. There was a delay of about 2 minutes between the FTIR record and TG results because it required time for gas to fill the spectrometer. All of the experiments were repeated at least three times to achieve a good repeatability.

### 2.3. Kinetic Reaction Scheme

In our previous study [21], MDF was considered as being comprised of resin, hemicellulose, cellulose, and lignin. Each component reacts as an *n*th order Arrhenius-type reaction: ${\mathrm{Component}}_{i}\to \gamma_{i}Tar+\delta_{i}Gases+\sigma_{i}Char,$ *step 1*; Tar→τGases+θChar, *step 2*, where *step 1* represents the primary pyrolysis, and *step 2* denotes the secondary charring reaction; *γ_i_*, *δ_i_* and *σ_i_* indicate the yield coefficient of tar, gases and char for component *i*, respectively. *τ* and *θ* refer to as the yield coefficient of gases and char for tar, respectively. Based on the scheme, the associate equations can be expressed as follows:

The reaction rates and decomposition rates for reactions in *step 1* can be expressed by

(1) $$\frac{dW_{i}}{dt}=-A_{i}exp\left(-\frac{E_{i}}{RT}\right)W_{i,0}{\left(\frac{W_{i}}{W_{i,0}}\right)}^{n_{i}},$$

(2) $$\frac{d\alpha_{i}}{dt}=A_{i}exp\left(-\frac{E_{i}}{RT}\right){\left(1-\alpha_{i}\right)}^{n_{i}},$$

where *W_i_* and *W_i,0_* represent the instantaneous and the initial mass fraction of component *i*, respectively. *A_i_*, *E_i_* and *n_i_* denote the pre-exponential factor, activation energy and reaction order of the reactions of component *i*, respectively; *R* stands for the gas constant; *α_i_* indicates the conversion rate which can be described as

(3) $$\alpha =\frac{m_{0}-m_{t}}{m_{0}-m_{\infty }},$$

where *m_0_*, *m_t_*, and *m_∞_* refer to the initial, instantaneous and the final solid mass of samples, respectively.

Thus, the mass loss rate *(dW/dt)_1_* and the solid mass fraction *W_1_* of *step 1* can be estimated as follows:

(4) $${\left(\frac{dW}{dt}\right)}_{1}=\overset{n}{\underset{i}{\sum }}(\frac{dW_{i}}{dt}+\frac{dW_{char,i}}{dt})=\overset{n}{\underset{i}{\sum }}(\frac{dW_{i}}{dt}+\sigma_{i}W_{i,0}\frac{d\alpha_{i}}{dt}),$$

(5) $$W_{1}=1-\sum_{i}^{n}\left(1-\sigma_{i}\right)\alpha_{i}W_{i,0}.$$

The reaction rate for secondary charring reaction can be evaluated by

(6) $$\frac{dW_{tar}}{dt}=-A_{tar}exp\left(-\frac{E_{tar}}{RT}\right)W_{tar,0}{\left(\frac{W_{tar}}{W_{tar,0}}\right)}^{n_{tar}},$$

(7) $$\frac{d\alpha_{tar}}{dt}=A_{tar}exp\left(-\frac{E_{tar}}{RT}\right){\left(1-\alpha_{tar}\right)}^{n_{tar}},$$

where *W_tar,0_* represents the initial mass fraction of tar which is calculated from *step 1*:

(8) $$\frac{dW_{tar,0}}{dt}=\sum_{i}^{n}\gamma_{i}W_{i,0}\frac{d\alpha_{i}}{dt}.$$

Therein, *W_tar_*, *A_tar_*, *E_tar_*, *n_tar_* denote the instantaneous mass fraction, the pre-exponential factor, the activation energy and the reaction order of reaction for tar generated by *step 1*, respectively. 

Thus, the mass of char generated by *step 2 W_char,2_* is calculated as

(9) $$\frac{dW_{char,2}}{dt}=\theta W_{tar,o}\frac{d\alpha_{tar}}{dt}.$$

Therefore, the total solid mass fraction in the whole process can be expressed as

(10) $$W_{tot}=W_{1}+W_{char,2}.$$

The total mass loss rate of whole process is then deduced as

(11) $${\left(\frac{dW}{dt}\right)}_{tot}=\sum_{i}^{n}(\frac{dW_{i}}{dt}+\sigma_{i}W_{i,0}\frac{d\alpha_{i}}{dt})+\theta W_{tar,o}\frac{d\alpha_{tar}}{dt}.$$

The yields of pyrolysis products can be predicted by the equations above. The yield of char can be expressed as

(12) $$W_{char}=\sum_{i}^{n}\sigma_{i}W_{i,0}\alpha_{i}+\theta W_{tar,o}\alpha_{tar}.$$

The yield of gas can be calculated by

(13) $$W_{gas}=\sum_{i}^{n}(1-\sigma_{i}-\gamma_{i})W_{i,0}\alpha_{i}+(1-\theta )W_{tar,o}\alpha_{tar}.$$

The yield of tar is calculated by the solid mass fraction and gas mass fraction:

(14) $$W_{tar}=1-W_{tot}-W_{gas}.$$

### 2.4. Optimization Technique—Differential Evolution Algorithm

When analyzing kinetic problems of biomass pyrolysis, two traditional methods—model-free and model-fitting—are usually involved to estimate kinetic parameters. However, when the reaction scheme above is applied, it is difficult to address the problem by traditional methods because of the large number of unknown parameters. Therefore, some optimization methods are used. Differential evolution (DE) is a sort of global optimization algorithm which has been applied in various research fields [24]. Assuming there are *M* individuals in a random population, which can be expressed as $X_{i}=\left(x_{i,1},x_{i,2},\ldots x_{i,n}\right)$, each individual undergoes initialization, variation, crossover and selection operations to determine the optimized value [25]. The implementation of DE in the MATLAB program is one-to-one correspondence with the steps of the algorithm. 

**1:** Define constants. The constants in the algorithm should be defined, including the population size, crossover probability, scaling factor and the maximum iteration number.

**2:** Initialization. The initial value of the individual *X_i_* is calculated by

(15) $$X_{i}\left(g\right)=X_{i}^{L}+rand\left(0,1\right)\left(X_{i}^{U}-X_{i}^{L}\right),$$

where $X_{i}^{U}$,$X_{i}^{L}$ refer to the upper and lower bands of *X_i_*, respectively, rand (0, 1) denotes a random number distributed in [0, 1], and *g* indicates the number of iteration.

**3:** Variation. A variation individual is determined by

(16) $$V_{i}\left(g\right)=X_{r1}+F\left(X_{r2}-X_{r3}\right),$$

where *F* indicates the scaling factor, and *X_r1_, X_r2_, X_r3_* represent three different random numbers that are different to *X_i_*.

**4:** Crossover. The individual is updated via

(17) $$X_{i}^{\prime }\left(g\right)=\{\begin{array}{cc} V_{i}\left(g\right), & rand\left(0,1\right)\leq Cr\\ X_{i}\left(g\right), & otherwise \end{array},$$

where *Cr* denotes the crossover probability.

**5:** Selection. The individual in the next iteration is selected by

(18) $$X_{i}\left(g+1\right)=\{\begin{array}{cc} X_{i}^{\prime }\left(g\right), & f\left(X_{i}^{\prime }\right)<f\left(X_{i}\right)\\ X_{i}\left(g\right), & otherwise \end{array},$$

where *f(x)* denotes the fitness function of the individuals. In this study, the fitness function *φ* is the error between model outputs and experimental results, which can be calculated as

(19) $$\varphi =\sum_{1}^{k}c\frac{\sum_{1}^{n_{j}}\left|W_{cal,j}-W_{exp,j}\right|}{\sum_{1}^{n_{j}}W_{exp,j}}+\sum_{1}^{k}\left(1-c\right)\frac{\sum_{1}^{n_{j}}\left|{\left(\frac{dW}{dt}\right)}_{cal,j}-{\left(\frac{dW}{dt}\right)}_{exp,j}\right|}{\sum_{1}^{n_{j}}{\left(\frac{dW}{dt}\right)}_{exp,j}},$$

where *cal* denotes the calculated results, *exp* refers to the corresponding experimental data, *n_j_* indicates the number of data points and *k* represents the number of heating rates. In order to make the weight of mass and mass loss rate equal, the weight coefficient *c* is set to 0.5. In respect to the algorithm parameters, population size, crossover probability, scaling factor and maximum iteration number are set as 60, 0.2, 0.5 and 1000 in this work, respectively. The update process is repeated until a satisfactory solution is obtained.

## 3. Results and Discussion

### 3.1. Thermogravimetric Analysis

Some characteristic parameters observed in mass fraction (TG)-mass loss rate (DTG) curves are shown in Table 3. *T_i_* indicates the initial temperature of the primary pyrolysis stage, *T_max_* represents the temperature corresponding to the maximum mass loss rate, *T_f_* denotes the terminal temperature of the primary pyrolysis stage and *t_max_* refers to the time corresponding to the maximum mass loss rate.

#### 3.1.1. Effects of Water Washing on Weight Loss

**Figure 1:**
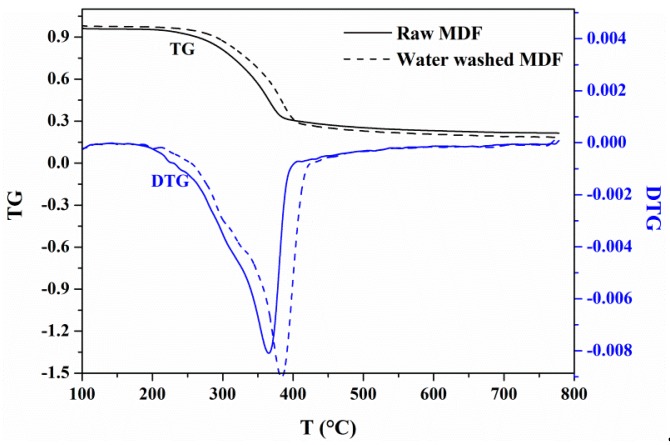
TG and DTG profiles of raw MDF and water washed MDF.

The mass fraction (TG) and mass loss rate (DTG) profiles of raw MDF and water washed MDF are compared in Figure 1. It shows that both the TG and DTG curve of washed MDF shifted to a high temperature. As indicated in Table 3, the primary pyrolysis stage of raw MDF started at 182.6 °C, reached its maximum mass loss rate at 365.3 °C and ended at 395.1 °C. After water washing, the initial and ending primary pyrolysis stage temperatures were 192.5 °C and 416.5 °C, respectively, indicating that the primary pyrolysis stage started later and became wider after water washing. In addition, the final char yield of raw MDF was shown to be higher than that of water washed MDF. Analyzing these differences, it can be concluded that the minerals play a positive role in decomposition and char formation of MDF pyrolysis.

#### 3.1.2. Effects of KCl on the Weight Loss Profile

**Figure 2:**
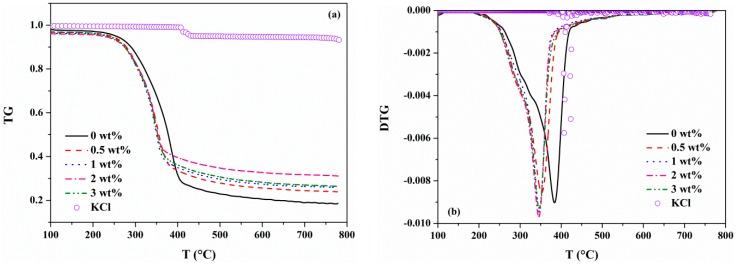
TG (**a**) and DTG (**b**) profiles of pure KCl sample and of MDF samples with different KCl loading concentrations.

Figure 2 shows the TG-DTG curves of MDF samples with varying KCl loading concentrations. The TG-DTG curves of pure KCl are also presented. It can be seen that the mass of pure KCl exhibited a slight loss above 400 °C. Therefore, there was little influence of the degradation of KCl on the MDF samples since the maximum weight percent of KCl added to the MDF samples was 3 wt%. As shown in Figure 2a, the main mass loss of 0 wt% KCl sample started at 192.5 °C, reached its maximum value at 383.5 °C and ended at 416.5 °C. Then, the mass loss slightly decayed until 800 °C. The former stage corresponding to the primary pyrolysis occurred at lower temperatures, whereas the latter corresponded to the secondary pyrolysis stage [26]. When MDF was doped with KCl, there was a notable change to the characteristic parameters. The final temperatures were reduced as the KCl concentration increased, whereas KCl had a much weaker influence on the initial temperatures. The temperature range for the primary stage of MDF pyrolysis was narrowed by almost 24–36 °C. In addition, *T_max_* decreased with the increasing concentration of KCl, from 383.5 °C of 0 wt% KCl to 345.7 °C of 3 wt% KCl. This revealed that the presence of KCl makes the pyrolysis reach the maximum mass loss rate in advance, and thus potassium had a catalytic effect on the MDF pyrolysis [22,27,28,29]. The final char yields of the different samples doped with 0, 0.5, 1, 2 and 3 wt% KCl were 18.6%, 23.9%, 25.9%, 31.1% and 26.4%, respectively. This suggests that the presence of KCl promoted the formation of char. Similar results were obtained from previous studies [8,22,30]. It is worth noting that when the addition of KCl increased to 3 wt%, the characteristic temperatures changed slightly and the final char yield was less than 2 wt% KCl sample. The most likely reason was that excessive KCl might have a negative effect on the mass and heat transfer, thus influencing the pyrolysis products [31,32].

### 3.2. FTIR Analysis

**Figure 3:**
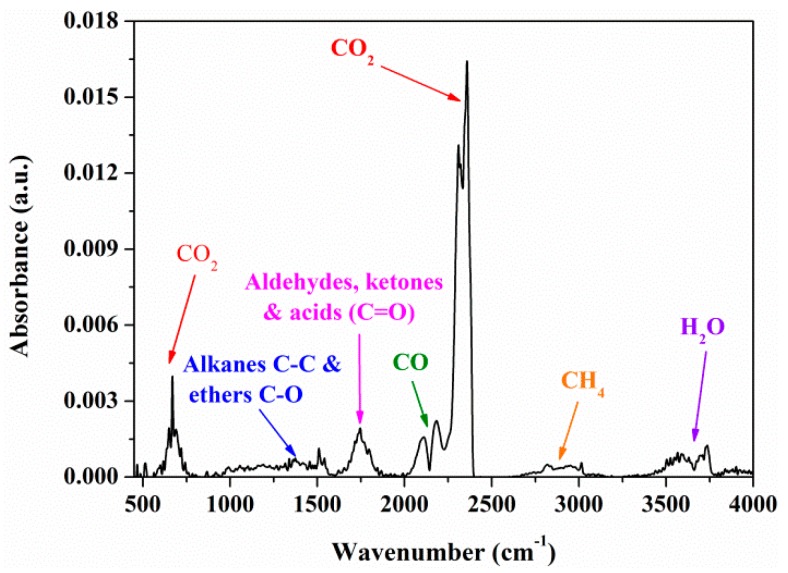
Characteristics absorbance of gaseous pyrolysis products of raw MDF at *T_max_*.

**Figure 4:**
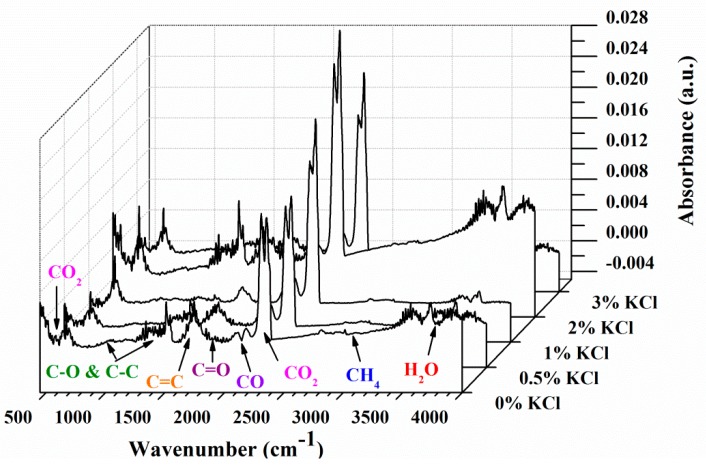
FTIR analysis of MDF samples with different KCl loading concentrations at *T_max_*.

FTIR was involved to characterize the gases the escaped in the pyrolysis process of MDF samples. With the temperature fixed at *T_max_*, the strongest characteristic absorbance can be obtained. Some possible compounds from FTIR characteristics absorbance bands, as summarized in Table 4 [33,34,35,36,37,38], were observed. Figure 3 shows the characteristics of absorbance of gaseous pyrolysis products of raw MDF at *T_max_*. The main gases released during raw MDF pyrolysis were obtained based on Table 4. Figure 4 indicates the absorbance at various wavenumbers of MDF samples with varying KCl loading concentrations at *T_max_*. It was observed that the presence of KCl had effects on the amounts of pyrolysis gaseous products instead of the composition, which is consistent with the result reported by Feng et al. [8].

According to the Lambert−Beer law, the gas concentration is linearly dependent on the absorbance at a specific wavenumber [39,40]. Therefore, the trends in the concentrations of gas products can be analyzed by the variations in the absorbance. As demonstrated in Figure 3, the absorbance peak at 3014 cm^−1^ corresponded to the release of methane (CH_4_). The absorbance peak at 2358 cm^−1^ was attributed to the release of carbon dioxide (CO_2_). The absorbance peak at 2182 cm^−1^ indicated the release of carbon monoxide (CO). The absorbance peak at 1748 cm^−1^ represented the release of aldehydes, ketones and acids (C=O). The absorbance at the corresponding wavenumber were normalized to evaluate the effect of KCl concentration on pyrolysis gas products CH_4_, CO_2_, CO and C=O.

**Figure 5:**
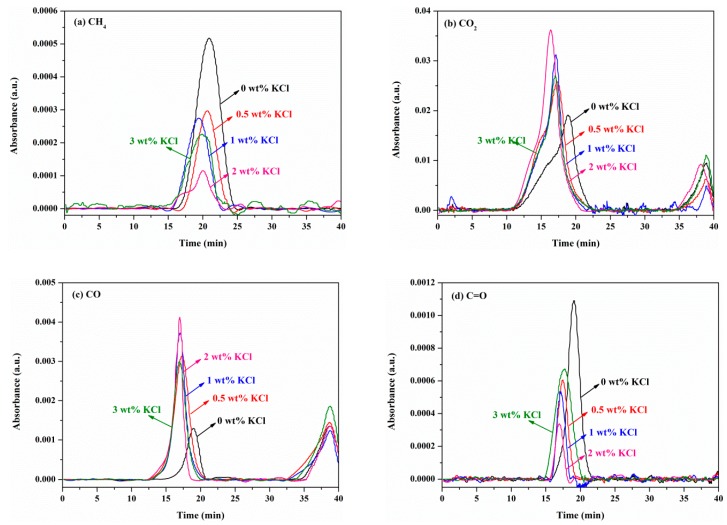
Changes in absorbance of gas products of MDF samples with different KCl concentrations with time: (**a**) CH4; (**b**) CO2; (**c**) CO; and (**d**) C=O.

Figure 5 shows the changes in absorbance of gas products of the MDF sample with varying KCl concentrations with time. The presence of KCl significantly affected the release of CH_4_, CO_2_, CO and C=O. For each gas product, the evolution profile shifted to an earlier time with the KCl concentration, which was consistent with the trends of *T_f_* shown in Table 3. Similar to the findings in TG-DTG curves above, the trend of 3 wt% KCl remained an exception. For CH_4_ and C=O compounds, the peak absorbance dropped with the increase in KCl concentrations. On the contrary, as shown in Figure 5b, the peak absorbance of CO_2_ increased when the concentration of KCl increased. The trend of CO peak absorbance was similar to that of CO_2_. In addition, relatively high amounts of CO_2_ were released for each sample. Meanwhile, the release of CH_4_ was the minimum. As shown in Figure 5, the peak absorbance of all gas products appeared at low temperatures (early time), while CO_2_ and CO showed another peak at higher temperatures. It implies that there was more CO_2_ and CO evolved in the secondary pyrolysis stage [8,26].

### 3.3. Kinetic Analysis

#### 3.3.1. Model Parameters Optimization

In previous research [21], the process of estimating the optimization value of model parameters was introduced in detail. Put simply, an appropriate search range of each parameter should be clarified, and then the DE algorithm should be applied to estimate the optimal value of for every single parameter. When performing a calculation of the fitness function *φ* in Equation (19), the experimental data from heating rates of 20 and 30 °C/min were utilized. Making an adjustment of the search ranges for parameters listed in [21], the optimized values of model parameters of different MDF samples were obtained and listed in Table 5.

**Figure 6:**
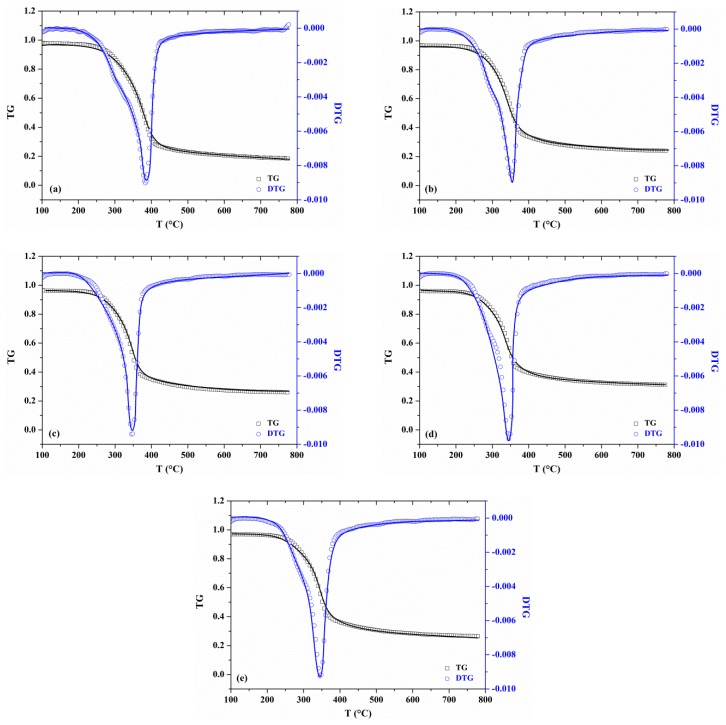
Comparisons between experimental TG/DTG data and predictions based on optimized parameters of different MDF samples at heating rate of 20 °C/min: (**a**) 0 wt%; (**b**) 0.5 wt%; (**c**) 1 wt%; (**d**) 2 wt%; (**e**) 3 wt%. Experimental data are shown in scatters; prediction results are shown in solid lines.

Comparisons between the experimental TG/DTG data and calculated values of MDF samples with varying KCl loading concentrations at the heating rate of 20 °C/min are presented in Figure 6. An excellent consistency between predicted results and experimental data are observed for both TG and DTG curves. These optimized parameters can satisfy not only the heating rates of 20 °C/min but also the heating rate of 30 °C/min. Therefore, the optimized parameters obtained can be validated as appropriate for other heating rates.

#### 3.3.2. Effects of KCl on MDF Components and Pyrolysis Products

**Figure 7:**
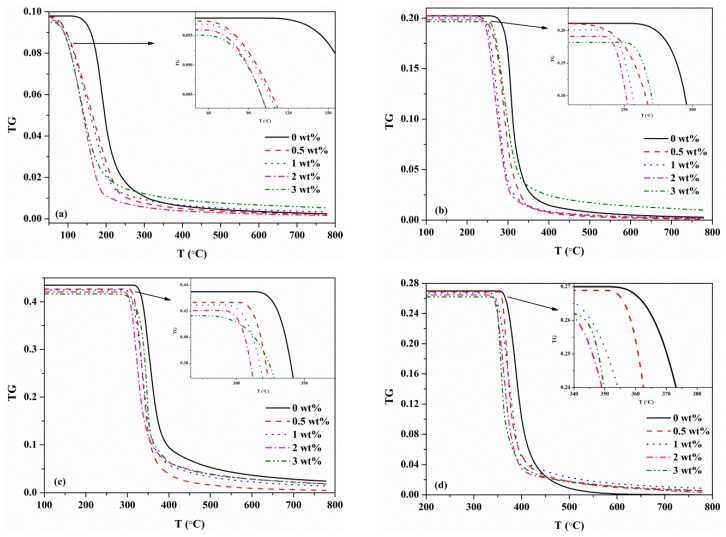
Comparisons of components mass fraction at different KCl loading concentration: (**a**) resin; (**b**) hemicellulose; (**c**) cellulose; (**d**) lignin.

Figure 7 shows the predicted TG curves of each component for different MDF samples. According to the analysis above, when the KCl was increased to 3 wt%, the trends of TG and the absorbance of gas products were discovered to be inconsistent with other KCl loading concentrations. A similar phenomenon can be observed in Figure 7. Thus, the effects of KCl on MDF components and pyrolysis products were analyzed without the case of 3 wt% KCl. Obviously, the initial mass fraction of each component decreased as KCl increased. A trend of mass fraction decreasing with the temperature was spotted for each component. For resin, when the addition of KCl increased, the temperature at which the resin started to decompose decreased. That is, the initial decomposition temperature shifted forward, from 172 °C of 0 wt% KCl to 100 °C of 2 wt% KCl. Similarly, as can be seen from Figure 7b−d, the initial decomposition temperatures of hemicellulose, cellulose and lignin moved forward as the KCl concentration increased. The initial degradation temperatures of these three components dropped from 300 °C, 354 °C and 370 °C of 0 wt% KCl to 248 °C, 310 °C and 357 °C of 2 wt% KCl, respectively. In contrast to hemicellulose and cellulose, KCl had a relatively weak effect on the initial degradation temperature of lignin. Therefore, it can be predicted that the addition of potassium increases the reaction rates of resin, hemicellulose and cellulose. In comparison, the catalytic effect of potassium on the reaction of lignin was relatively weak. The analysis about cellulose conforms to the study performed by Trendewicz et al. [16].

**Figure 8:**
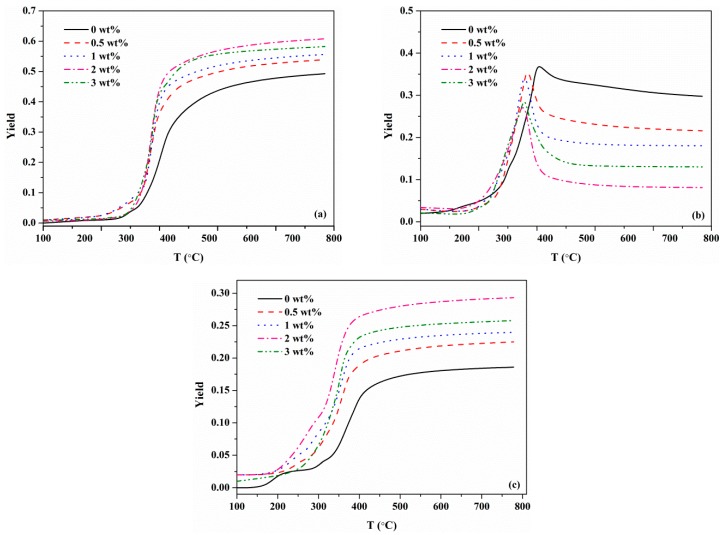
Comparisons of pyrolysis product yields at different KCl loading concentrations: (**a**) gas; (**b**) tar; (**c**) char.

Figure 8 shows the predicted yields of pyrolysis products of different MDF samples. Obviously, the yields of char and gas increased with the concentration of KCl. Conversely, the yield of tar reduced. This phenomenon was found to be consistent with the study conducted on the effect of KCl on cellulose pyrolysis products in [22]. Aside from this, as the KCl concentration increased, the temperature at which the pyrolysis products reached their respective maximum yields decreased. For tar, it was observed from Figure 8b that the temperatures corresponding to the peak yields were about 416 °C, 391 °C, 383 °C and 379 °C of samples with 0 wt%, 0.5 wt%, 1 wt% and 2 wt% KCl. Moreover, these temperatures were consistent with *T_f_* in Table 3, indicating that tar yield reached its peak value at the end of the primary pyrolysis. When the temperature was above *T_f_*, there would be a reduction in tar yield curve. This was most likely when *T* > *T_f_*, the secondary charring reaction occurred, and the generation rate of tar in primary pyrolysis was low, as compared with the consumption rate of tar in the secondary reaction.

**Figure 9:**
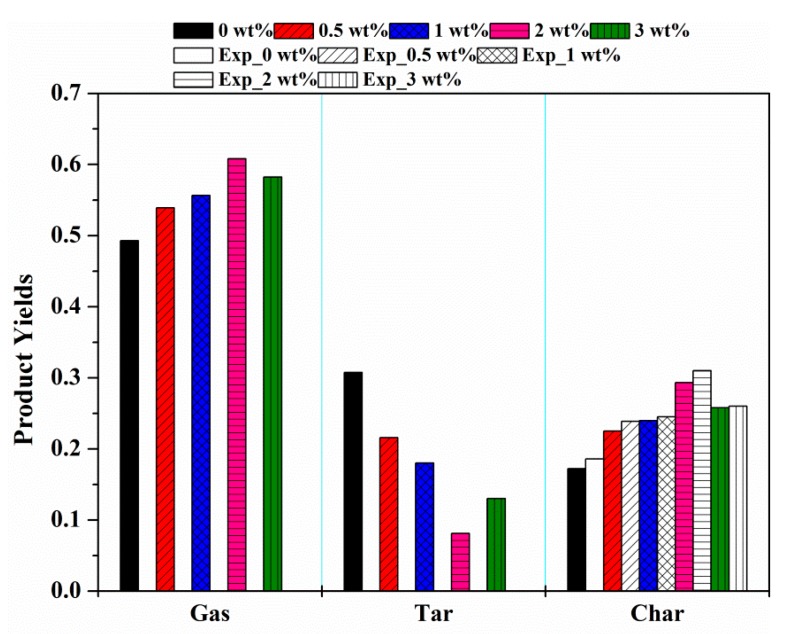
Comparisons of pyrolysis product yield at the final temperature.

Figure 9 shows a comparison of the yields of pyrolysis products at the final temperature of 800 °C. The addition of KCl increased the yields of gas and char obviously. For gas, the yield increased from 49.3% of 0 wt% KCl to 60.8% of 2 wt% KCl. For char, the yield increased from 17.2% of 0 wt% KCl to 29.3% of 2 wt% KCl. On the contrary, tar yield was reduced from 30.8% of 0 wt% KCl to 8.1% of 2 wt% KCl. The experimental char yield could be determined by weighing the residue amount after the experiment [16,31]. In comparison with the predicted char yield with the experimental data, the experimental char yield was slightly larger than the predicted value. The most probable reason was that the experimental char yield was actually the residual solid mass, including the unreacted mass of each component.

#### 3.3.3. Effects of KCl on Activation Energies

In order to find out the underlying reason of the observed changes in MDF components and pyrolysis products due to potassium, the changes in activation energies of primary reactions and secondary charring reaction were analyzed.

**Figure 10:**
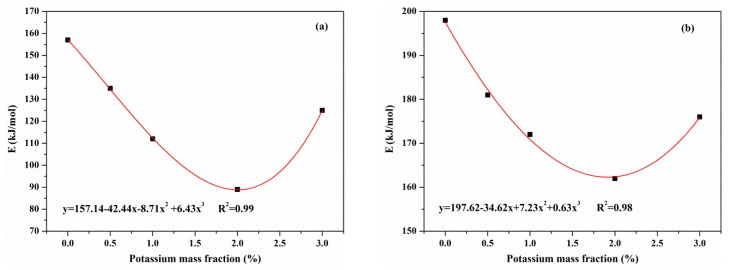

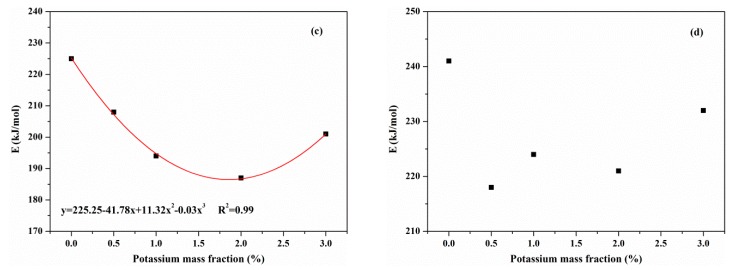
The effect of potassium concentration on activation energy of (**a**) resin; (**b**) hemicellulose; (**c**) cellulose; (**d**) lignin in primary reactions.

**Figure 11:**
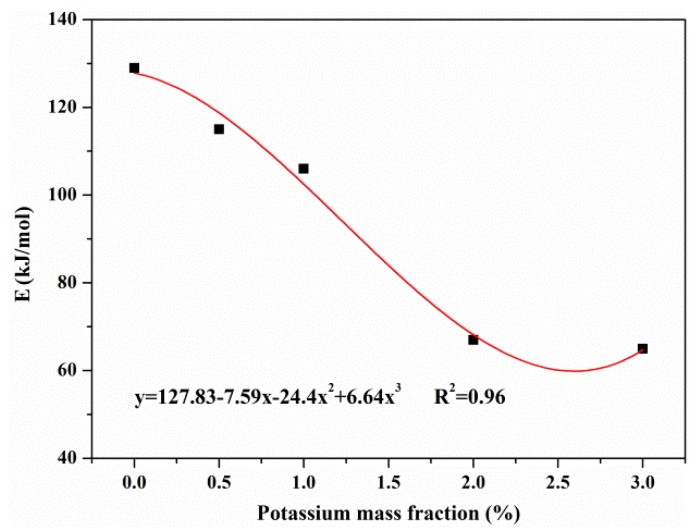
The effect of potassium concentration on activation energy of secondary charring reaction.

Figure 10a−d shows the activation energies for the reactions of resin, hemicellulose, cellulose and lignin change with potassium concentrations, respectively. Figure 11 indicates the activation energy of the secondary charring reaction change with potassium concentrations. As the activation energies of reactions of resin, hemicellulose, cellulose and tar vary depending on the potassium treatment mass fraction (%), by fitting the evaluated data presented in Figure 10 and Figure 11, some functions are derived:

(20) $$E_{r}=157.14-42.44x-8.71x^{2}+6.43x^{3},\;R^{2}=0.99,$$

(21) $$E_{h}=197.62-64.62x+7.23x^{2}+0.63x^{3},\;R^{2}=0.98,$$

(22) $$E_{c}=225.25-41.78x+11.32x^{2}-0.03x^{3},\;R^{2}=0.99,$$

(23) $$E_{tar}=127.83-7.59x-24.4x^{2}+6.64x^{3},\;R^{2}=0.96,$$

where *E* represents the activation energy, and *r, h, c* denote the reactions of resin, hemicellulose and cellulose in primary pyrolysis, respectively. *tar* denotes the secondary charring reaction and x indicates the mass fraction of potassium (%). 

As demonstrated in Figure 10, when the potassium mass fraction was less than 2 wt%, with the increasing of the addition of KCl, the activation energy of the reaction of resin reduced, indicating that the energy required for resin reaction was reduced. Similarly, the activation energies of reactions of hemicellulose and cellulose reduced with the KCl concentration. However, there was yet to be a consistent trend shown between the KCl concentration and activation energy of lignin reaction. The potassium had little effect on the activation energy of lignin. The most probably reason was that the potassium had little influence on the generation of free radicals which was the main reaction to occur during the lignin pyrolysis process [31]. Thus, the addition of potassium catalyzed the reaction of resin, hemicellulose and cellulose, but it had little effect on the reaction of lignin.

According to the fitting curve shown in Figure 11, when the potassium mass fraction was less than approximately 2.5 wt%, the activation energy of the secondary charring reaction decreased with the concentration of KCl. Potassium thus played a catalytic role in the reaction of tar. Therefore, the catalytic effect of KCl on MDF pyrolysis was the combination of the primary pyrolysis and the secondary charring reaction.

In this work, the impact of 3 wt% KCl was an extraordinary one. When the addition of KCl increased to 3 wt%, the trends of characteristic parameters, including the initial decomposition temperature of each component, the yields of products and the activation energies of pyrolysis reactions, were found to be distinct from other cases. A limit on the concentration of KCl may exist to achieve the best catalytic effects on MDF pyrolysis. Based on the fitting curves shown in Figure 10 and Figure 11, it is reasonable to conclude that 2 wt% and 2.5 wt% are the optimal concentrations of KCl for the catalytic effects exerted on MDF pyrolysis in the primary pyrolysis and secondary charring reaction, respectively. 

## 4. Conclusions

In the present study, five MDF samples treated with varying potassium chloride (KCl) concentrations were heated by thermogravimetry/Fourier-transfer infrared spectroscopy (TG-FTIR) to investigate the effects of potassium chloride on the pyrolysis of medium density fiberboard (MDF). The kinetic parameters were estimated through a procedure based on experimental data and a differential evolution optimization algorithm. The major results and conclusions are summarized as follows:

(1) The presence of KCl shifted the primary pyrolysis stage towards lower temperatures. As the addition of KCl increased, the characteristic initial temperature showed little change while the final temperature decreased. The temperature range of the primary pyrolysis process was narrowed by nearly 24−36 °C. The mass loss rate of the whole pyrolysis process reached its peak in advance with the concentration of KCl.
(2) KCl affected the release of pyrolysis gaseous products in terms of amounts instead of composition. KCl inhibited the formation of CH_4_ and C=O functional groups while promoting the formation of CO_2_ and CO.
(3) KCl changed the initial degradation temperature for each component of MDF. With the increasing concentration of KCl, the initial degradation temperature of resin, hemicellulose and cellulose dropped. For lignin, the effect of KCl on the initial degradation temperature was relatively weak.
(4) Char and gas yields increased with the increasing concentration of KCl, whereas the tar yield was reduced.
(5) KCl had an effect on the activation energies of reactions during the pyrolysis process. In the primary pyrolysis stage, the activation energies of resin, hemicellulose and cellulose decreased with the concentration of KCl. Aside from this, KCl had little effect on the activation energy of lignin. In the secondary charring reaction, KCl reduced the activation energy of tar. The catalytic effect of KCl on MDF pyrolysis manifested in the combination of the primary pyrolysis and secondary charring reaction.
(6) It is reasonable to take 2 wt% and 2.5 wt% as the optimal concentrations of KCl for the catalytic effects on MDF pyrolysis in primary pyrolysis and the secondary charring reaction, respectively.

## Figures and Tables

**Table 1:** Proximate and ultimate analysis of raw medium density fiberboard (MDF) sample.

Proximate Analysis (wt%)					Ultimate Analysis (wt%)					
Moisture	Ash	Volatiles	Fixed Carbon	error	C	H	O^a^	N	S	error
8.28	4.69	81.2	5.83	0.98%	44.96	6.26	44.87	3.35	0.56	1.48%

^a^ Calculated by difference.

**Table 2:** Composition analysis of raw MDF ash and water washed MDF ash.

Samples	Ash Chemical Compositions (wt%)										
	Ca	K	S	Si	Mg	P	Al	Fe	Na	Cl	Mean Error
Raw MDF	22.87	10.66	3.45	3.11	2.52	1.53	1.09	0.91	0.62	0.53	1.82%
Water washed MDF	18.01	1.25	1.64	6.41	1.73	0.48	2.62	1.29	1.19	0.20	1.46%

**Table 3:** Characteristic parameters of the mass fraction (TG) and mass loss rate (DTG) curves.

Sample	T_i_ (°C)	T_max_ (°C)	T_f_ (°C)	t_max_ (min)
raw MDF	182.6	365.3	395.1	18.6
0 wt% KCl (water washed MDF)	192.5	383.5	416.5	19.5
0.5 wt% KCl	192.4	352.0	391.5	17.9
1 wt% KCl	191.4	347.1	383.7	17.7
2 wt% KCl	191.3	345.3	379.9	17.6
3 wt% KCl	188.9	345.7	387.1	17.6

**Table 4:** A summary of the possible compounds from characteristics absorbtion bands of FTIR.

Wavenumbers, cm^−1^	Chemical Bond	Vibrations	Compounds
4000−3400	O−H	stretching	H_2_O
3050−2650	C−H	stretching	CH_4_
2400−2240	C=O	stretching	CO_2_
2230−2000	C−O	stretching	CO
1880−1620	C=O	stretching	aldehydes, ketones, acids
1600−1420	C=C	stretching	aromatics
1300−950	C−O, C−C	stretching	alkanes & ethers
750−560	C=O	bending	CO_2_

**Table 5:** Optimized values of kinetic parameters for different KCl loading concentrations.

Component	Parameter	Range	0 wt% KCl	0.5 wt% KCl	1 wt% KCl	2 wt% KCl	3 wt% KCl
Resin	ln*A_r_* (ln s^−1^)	(2.4, 47.6)	21.82	24.34	20.9	24.12	22
	*E_r_* (kJ/mol)	(6.9, 200)	157.5	136.56	112	89	125.3
	*n_r_*	(0.1, 8)	2.05	2.07	2.2	2	2.5
	*W_r,o_* (%)	(1, 20)	9.80	9.75	9.7	9.6	9.51
	*φ_r_/σ_r_* (%)	(5, 95)	31.8	42	23.65	58.32	53.21
	*γ_r_* (%)	(5, 95)	7.0	8.78	21.36	16.85	8.7
Hemicellulose	ln*A_h_* (ln s^−1^)	(3.1, 62.4)	32	33.04	39.95	29.96	33.9
	*E_h_* (kJ/mol)	(11.3, 225.3)	198	181	172.35	162.56	176
	*n_h_*	(0.1, 8)	1.9	1.7	1.89	1.78	1.49
	*W_h,o_* (%)	(1.8, 36)	20.23	20.22	20.03	19.83	19.64
	*φ_h_/σ_h_* (%)	(5, 95)	10.4	16.7	21.46	11.36	17.58
	*γ_h_* (%)	(5, 95)	49.5	48.3	61.1	50.32	30.36
Cellulose	ln*A_c_* (ln s^−1^)	(3.9, 78.2)	35	40.4	38.06	35.69	39.4
	*E_c_* (kJ/mol)	(16, 319.4)	225	208.65	194.63	187.53	201.64
	*n_c_*	(0.1, 8)	2.5	1.9	2.26	2.41	2.42
	*W_c,o_* (%)	(4.8, 96)	42.9	42.68	42.48	42.06	41.65
	*φ_c_/σ_c_* (%)	(5, 95)	12.3	13.4	12.94	30.58	25.64
	*γ_c_* (%)	(5, 95)	42.9	27.2	43.85	52.25	28.33
Lignin	ln*A_l_* (ln s^−1^)	(5, 100.7)	39	35	39.01	42.24	38.83
	*E_l_* (kJ/mol)	(22.6, 451.8)	241	218	224.36	221.43	232.96
	*n_l_*	(0.1, 8)	1.4	2	2.24	1.82	2.27
	*W_l,o_* (%)		27	26.89	26.73	26.47	26.21
	*φ_l_ /σ_l_* (%)	(5, 95)	3.4	8.1	7.32	12.25	5.39
	*γ_l_* (%)	(5, 95)	69.2	78	73.25	63.02	38.65
Tar	ln*A_t_* (ln s^−1^)	(1.8, 35.8)	33.4	33.85	33.8	33.93	33.5
	*E_t_* (kJ/mol)	(12.4, 248)	129.3	115.85	106.65	67.74	65.69
	*n_t_*	(0.1, 8)	2.87	2.29	1.44	1.57	1.46
	*θ* (%)	(5, 95)	14.1	18.2	15.20	5.65	23.02
